# The Impact of Concave Coastline on Rainfall Offshore Distribution over Indonesian Maritime Continent

**DOI:** 10.1155/2019/6839012

**Published:** 2019-01-01

**Authors:** Furqon Alfahmi, Rizaldi Boer, Rahmat Hidayat, Perdinan, Ardhasena Sopaheluwakan

**Affiliations:** ^1^Department of Meteorology, Indonesian Agency for Meteorology Climatology and Geophysics, Jakarta 10720, Indonesia; ^2^Department of Geophysics and Meteorology, Bogor Agricultural University, Bogor 16680, Indonesia; ^3^Research and Development Center, Indonesian Agency for Meteorology Climatology and Geophysics, Jakarta 10720, Indonesia

## Abstract

Indonesian Maritime Continent has the second longest coastline in the world, but the characteristics of offshore rainfall and its relation to coastline type are not clearly understood. As a region with eighty percent being an ocean, knowledge of offshore rainfall is important to support activity over oceans. This study investigates the climatology of offshore rainfall based on TRMM 3B42 composite during 1998-2015 and its dynamical atmosphere which induces high rainfall intensity using WRF-ARW. The result shows that concave coastline drives the increasing rainfall over ocean with Cenderawasih Bay (widest concave coastline) having the highest rainfall offshore intensity (16.5 mm per day) over Indonesian Maritime Continent. Monthly peak offshore rainfall over concave coastline is related to direction of concave coastline and peak of diurnal cycle influenced by the shifting of low level convergence. Concave coastline facing the north has peak during northwesterly monsoonal flow (March), while concave coastline facing the east has peak during easterly monsoonal flow (July). Low level convergence zone shifts from inland during daytime to ocean during nighttime. Due to shape of concave coastline, land breeze strengthens low level convergence and supports merging rainfall over ocean during nighttime. Rainfall propagating from the area around inland to ocean is approximately 5.4 m/s over Cenderawasih Bay and 4.1 m/s over Tolo Bay. Merger rainfall and low level convergence are playing role in increasing offshore rainfall over concave coastline.

## 1. Introduction

Indonesian Maritime Continent (IMC) is an archipelago tropical country which has the second longest coastline in the world after Canada. The length of the coastline reaches 95,181 km and the numbers of islands are 16,056. Distribution of global and regional rainfall is affected by IMC's coastline. The distribution of local rainfall amount (a rainfall point less than 300 km from coastline) is a gradually decreasing as function of coastal distance, while total regional rainfall (rainfall area more than 10,000 km^2^) is increasing as function of coastline density (coastal length/land area) [1].

The shape and size of the coastline over IMC are unique. Basically, there are three shapes of coastline which affect the rainfall amount, straight coastline, concave coastline, and convex coastline [2, 3]. Every type of coastline over specific location has different impact to generate rainfall system over IMC. Sea breeze raises up convergence zone over a convex coastline and divergence zone over concave coastline. Otherwise, land breeze creates convergence over convex coastline and divergence zone over a concave coastline. Upward movement of air induced by convergence zone fostering the development of clouds brings out high rainfall intensity over coastline [4, 5].

The rainfall system over IMC is known as the coastal regime. It has characteristic of heavy rainfall over coastal area and dominant movement phase which is divided into two types, seaside and landside regime. The seaside coastal regime peak occurs from evening to morning of the next day and propagates to offshore area. The landside coastal regime has landward phase propagation with the peak occurring from morning to evening [6–9]. Diurnal peak of rainfall is influenced by the size of island. The peak of landside coastal rainfall occurs in the early afternoon on a narrow island while on a large island the peak of inland rainfall occurs on the latter [10].

Offshore rainfall is formed from the clouds which grow and develop over inland and move to the ocean. Offshore rainfalls over west Sumatera develop over inland and migrate to ocean until 400 km from coastlines with the average speed of migration being approximately 10 m/s [6]. The offshore rainfall over the Bay of Bengal extends its phase propagation up to several hundred kilometers from the coastline with a propagation speed of 15–20 m/s. Phase of rainfall creates a line which is parallel with the coastline of Bay of Bengal [11]. The offshore rainfall over Papua Island (New Guinea) is observed up to 600 km off the coast with phase of propagation speed being about 15 m/s [12]. The rainfall offshore mechanism over northern Borneo has been documented by Houze [13]. Offshore rainfall develops until 200 km from the coast. Speed of rainfall offshore propagation over Borneo depends on monsoonal flow regime. Phase speed propagation is about 3 m/s in the easterly monsoonal flow regime and 7 m/s in the westerly monsoonal flow regime [14].

Offshore rainfall over northern [13] and western of Borneo [15] is initiated by intensive wind convergence at the low level due to the strong offshore flow to the western coast during the nighttime. Strong offshore flow is obtained from convection activity that grows earlier in the afternoon and evening over Borneo Island. Convergence zone by land breeze also induces high offshore rainfall over Malacca strait [7] and western coast of Sumatera [16].

Several previous studies have described the characteristics and mechanism of the offshore rainfall over IMC without considering the shape and the effect of the coastline [6, 7, 13–16]. This study focuses on the elucidation of the offshore rainfall variability over concave coastline and the dynamical atmosphere has been caused by shape of concave coastline. The analysis and simulation of the dynamical atmosphere which induce high offshore rainfall are produced by WRF-ARW model.

## 2. Materials and Methods

### 2.1. Rainfall Observation

In order to detect the distribution and variability of offshore rainfall over concave coastline, we analyze Tropical Rainfall Measuring Mission (TRMM) 3B42 (version 7) rainfall product during 17-year observation from 1998 to 2015. The 3B42 product contains estimated rain rates (mm per hour) derived from a combination of infrared radiation (IR), passive microwave, and radar data from TRMM and IR data from geostationary satellites. The data is 3 hourly and covers the domain between 50°S and 50°N, with a horizontal resolution of 0.25° x 0.25°.

### 2.2. Dynamical Atmosphere Simulation

In order to examine the dynamical atmosphere inducing the offshore rainfall, we conduct the Weather Research and Forecast-Advanced Research Weather (WRF-ARW) v 3.3, based on the NCEP FNL (Final) Operational Global Analysis data used for initial and boundary conditions. NCEP FNL is a reanalysis product from the Global Data Assimilation System (GDAS) which continuously collects observational data from the Global Telecommunications System (GTS) and other sources.

**Figure 1:**
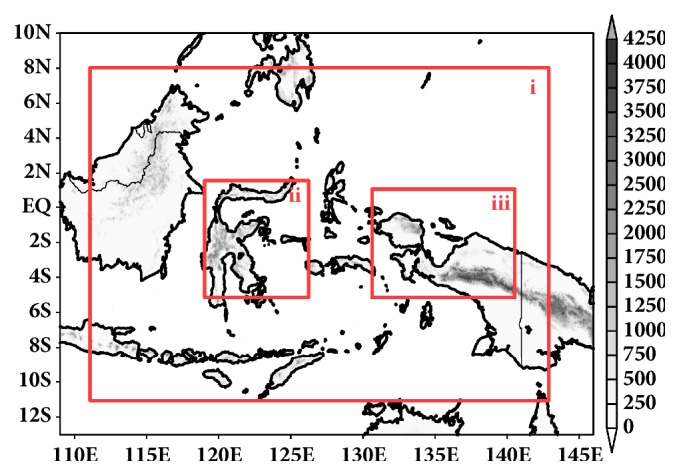
WRF-ARW nesting domain setting with (i) first domain resolution of 27 km, (ii) second domain resolution of 9 km representing Tolo Bay, and (iii) third domain resolution of 9 km representing Cenderawasih Bay.

In this experiment, we set up WRF-ARW with three nested configuration (see Figure 1). An outer domain has 133 × 81 grid points with a 27 km grid spacing, the second domain consists of 91 × 85 grid points with a 9 km grid spacing, and the third domain consists of 124 × 79 grid points with a 9 km grid spacing. The first domain is identic with the TRMM data resolution (≈ 0.25°). The second domain represents Cenderawasih Bay and the third domain represents Tolo Bay which is considered as coastline area curvature.

The microphysics WSM 6 class single-moment scheme by Hong and Lim [17] was selected for physical parameterizations including the planetary boundary layer scheme of Yonsei University Scheme (YSU) [18] and the Kain-Fritsch (KF) cumulus scheme [19] for this experiment. The KF scheme has three parts for calculation, first comparing parcel temperature with ambient temperature as the convective trigger function, second calculating the entrainment detrainment of water vapor as the mass flux formulation, and third rearranging mass in air column using the updraft, downdraft, and environmental mass fluxes until at least 90% of the convective available potential energy (CAPE) is removed as the closure assumptions. The land surface parameterization uses Noah LSM 4-layer. The WRF model was run at 42 vertical levels with the model top at 50 hPa. Details for all parameterization on this simulation are shown at Table 1.

Simulation of rainfall using WRF-ARW over IMC has been studied by Fujita [7], Bhatt [20], and Hassim [21]. Rainfall was produced by WRF-ARW which has overestimated the amplitude as over in land. But, it still captures the diurnal rainfall variability although it has coarse simulation with resolution of 10 km and 25 km. The utilization of the 25 km model resolution intended can explain meso-alpha scales phenomena, while the 10 km model explains meso-gamma scale features. The Kain-Fritsch convective scheme has a slight improvement compared to Betts-Miller-Janjic (BMJ) and Grell schemes [20].

## 3. Results and Discussion

### 3.1. Total Mean of Offshore Rainfall 

**Figure 2:**
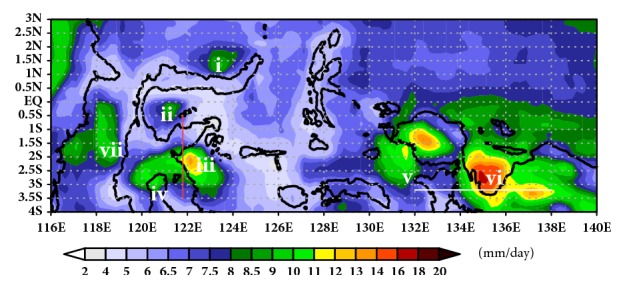
Total mean of offshore rainfall over Indonesian Maritime Continent based on composite TRMM during 1998 to 2015. The heavy offshore rainfall occurs over (i) Manado Bay, (ii) Tomini Bay, (iii) Tolo Bay, (iv) Bone Bay, (v), Berau Bay, (vi) Cenderawasih Bay, and (vii) Strait of Makassar. Red line cross section for Tolo Bay and white line cross section for Cenderawasih Bay.

The IMC has the greatest convective activity and the largest amount of rainfall in the world [22]. The existence interactions of seasonal and intraseasonal phenomena and complexity terrain in the archipelago island make the rainfall distribution more complex, both inland or offshore rainfall.  Figure 2 shows the complexity distribution of offshore rainfall over eastern part of IMC based on daily mean TRMM during 1998-2015. The offshore rainfall has increasing intensity just over concave coastline (e.g., Manado Bay, Tomini Bay, Tolo Bay, Bone Bay, Berau Bay, and Cenderawasih Bay) and strait area (e.g., Makassar Strait). Manado Bay, Tomini Bay, Tolo Bay, Bone Bay, and Berau Bay have intensity 9.1 mm per day, 9.0 mm per day, 13.5 mm per day, 10.5 mm per day, and 11.4 mm per day, respectively, while Strait of Makassar has intensity 9.3 mm per day. Cenderawasih Bay has highest intensity (16.5 mm per day) over IMC.

This pattern of offshore rainfall shows the greatness of the local effect. The peak of rainfall over concave coastline is located near the focus point of curvature line. Focus point is the point where sea breeze meets inducing the convergence zone with assumption that curvature has smooth line and the land breeze direction has perpendicular direction to the coastline. Because real coastline is irregular and unsmooth lines, the peak of offshore rainfall is shifted and irregularly shaped. Peak of offshore rainfall over Manado Bay and Tomini Bay is approximately 100–200 km from coastline while that over Tolo Bay, Bone Bay, and Cenderawasih Bay is very close to coastline. Previous research by Love [8] also explains the fact that amplitude of diurnal rainfall cycle over IMC is largest over the islands and surrounding coastal seas. In open ocean regions, where the distance is more than a few hundred kilometers from the large islands, the amplitude of the diurnal rainfall cycle is much smaller.

The strong or weak offshore rainfall intensity will be determined by the width of sea [7] and the surrounding mountains near the oceans [23, 24]. Fujita [7] explained that offshore rainfall over the Strait of Malacca (Sumatera Island) is higher than just over small area located in the northern part of the strait due to combined effect of width of sea and mountains. In farther north, the annual rainfall becomes smaller because the strait becomes wider. In farther south, the strait is more narrow; the offshore rainfall becomes smaller as well. It happens because there is no high mountain on each side of the strait in the southern part. There are ideal width of strait and ideal high terrain around strait which can induce morning offshore rainfall around the center of strait.

The effect of terrain height on land breeze was simulated by Qian [24]. His experiment revealed that terrain area creates stronger land breeze circulation as compared to the lower terrain case. The highest mountains around Cenderawasih Bay and Tolo Bay are approximately 3750 m (Mountain of Weyland) and 2590 m (Mountain of Pompangeo), respectively. Terrain high around Cenderawasih Bay is higher than Tolo Bay. The highest mountain around concave coastline combines the ideal width of concave coastline causing the offshore rainfall over Cenderawasih to be higher than another area.

**Figure 3:**
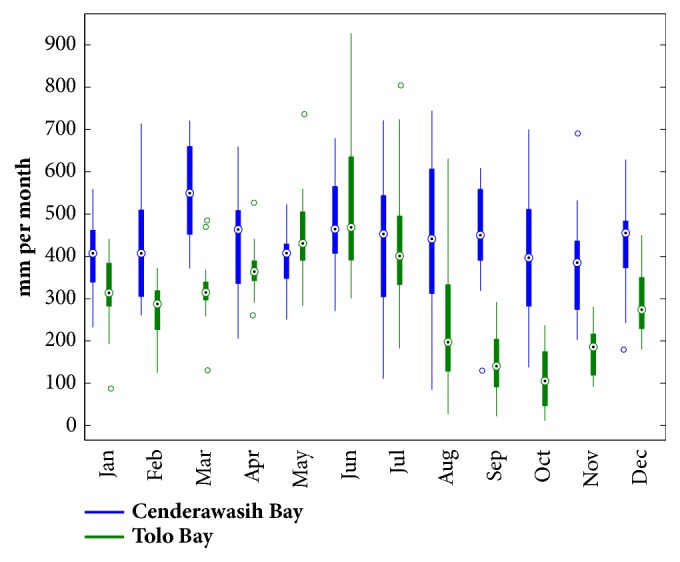
Box and whisker plot for monthly offshore rainfall variability over Cenderawasih Bay (blue) and Tolo Bay (red). The top and bottom of the large box denote the 25th and 75th percentiles. The circle in the box denotes the 50th percentile or median of the distribution. The bars extend to the largest or smallest value within 1.5 times the interquartile range of the 75th or 25th percentiles, respectively. The interquartile range is defined as the distance between the 25th and 75th percentile values. The whiskers are outliers.

Cenderawasih Bay and Tolo Bay are two of highest offshore rainfalls in Eastern Maritime Continent. For the next analysis, we use Cenderawasih Bay and Tolo Bay to reveal the characteristic of offshore rainfall over Eastern Indonesia Maritime Continent.  Figure 3 shows the peak of monthly offshore rainfall over Cenderawasih Bay and Tolo Bay.

Cenderawasih Bay has peak of offshore rainfall in March while Tolo Bay has peak of rainfall in June (see Figure 3). Nevertheless, Cenderawasih Bay tends to get wet every month because the offshore rainfall continues to occur throughout the year. Mean offshore rainfall over Cenderawasih Bay has 436.5 mm per month with minimum offshore rainfall being 371.5 mm per month and maximum offshore rainfall being 744.6 mm per month. Meanwhile, the range of offshore rainfall variability over Tolo Bay is higher than Cenderawasih Bay. During the dry season, offshore rainfall over Tolo Bay is lower than 22.2 mm per month, while during rainy season the offshore rainfall reaches 927.8 mm per month. Aldrian [25] groups Cenderawasih Bay as region A, while Tolo Bay is in the category of region C. Both region A and region C have a seasonal period rainfall peak but the time period is opposite. The peak of rainfall in region A occurs in the boreal winter, while region C is in the boreal summer.

The main factor of the different season of peak of offshore rainfall over Cenderawasih Bay and Tolo Bay is the direction of concave coastline faces. Because Cenderawasih Bay faces north, the northwesterly monsoonal flows on boreal winter will be blocked by the mountains and produce offshore rainfall on the north side of the mountains and over oceans. Conversely, because Tolo Bay faces east, the easterly monsoonal flows on boreal summer will be blocked by the mountains around Tolo Bay and produce offshore rainfall on Tolo water. The role of the mountain as a monsoonal wind block resulting in strong local rainfall around Cenderawasih Bay (Papua Island) is explained by Ichikawa [26] and Wu and Hsu [27] and around Tolo Bay (Sulawesi Island) is revealed by Assyakur [28].

### 3.2. Diurnal Offshore Rainfall 

**Figure 4:**
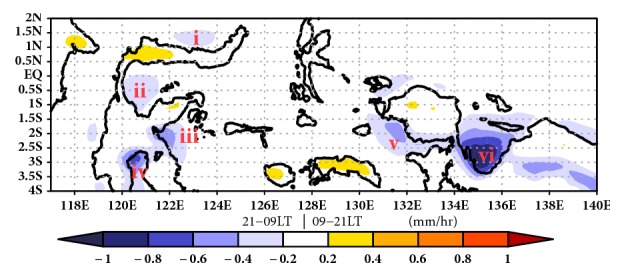
Different rainfall day times–nighttime around IMC. Minus value describes the rainfall mostly at nighttime over concave coastline, i.e., (i) Manado Bay, (ii) Tomini Bay, (iii) Tolo Bay, (iv) Bone Bay, (v) Berau Bay, and (vi) Cenderawasih Bay.

The rainfall cycle over IMC is predominant by diurnal variability. Nikita and Sekine [29] describe the fact that the diurnal rainfall cycle over IMC has maximum convective activity during the afternoon to evening hour over land, while maximum convectivity over ocean is from midnight to morning.  Figure 4 shows different offshore rainfall of daytimes and nighttime over IMC. The different offshore rainfall over concave coastline (e.g., Manado Bay, Tomini Bay, Tolo Bay, Bone Bay, Berau Bay, and Cenderawasih Bay) has minus value, while over island it has positive value. The minus value indicates that the offshore rainfall over concave coastline is dominated by nighttime convectivity.

The regional variation of annual mean difference between morning rainfall and evening rainfall over IMC observed by TRMM 3G68 has been examined by Mori [6]. The offshore rainfall over small concave coastline is not clearly visible on the map because it uses a sparse resolution. TRMM 3G68 has a resolution of 0.5° x 0.5°, while TRMM 3B42 has a higher resolution which is 0.25° x 0.25°. In contrast to inland region, offshore rainfall over western coast of Sumatera is approximately 60% of the rainfall amount that comes from morning rain for any rain types, and the convective rainfall fraction is 57% of the daily mean. Nearly similar to the offshore rainfall over western coast of Sumatera, offshore rainfall over Cenderawasih Bay is about 56% of the rain rate that comes from midnight to morning with the stratiform as the predominant cloud type. The midnight to morning rainfall has the northward propagation appearing at midnight in the northern coastal sea region of Papua Island and near Biak Island in the morning [30].

In order to examine the dynamical atmosphere which induces intensive offshore rainfall over the concave coastline, we use WRF-ARW to simulate the offshore rainfall and determine the wind variability. The study of comparing the rainfall over IMC was simulated by a 20 km grid Meteorological Research Institute General Circulation Model (MRI-GCM) and the near-surface rain data of TRMM 2A25 examined by Hara [31]. The feature of the rainfall generally agrees with the observation of the island and strait of having horizontal scales smaller than 200 km. For larger island and oceans, GCM fails to simulate the diurnal cycle. The rainfall simulation over IMC using WRF-ARW has been revealed by Bhatt [20]. All WRF-ARW simulations have overestimated the rainfall intensity over the land. The result suggests using higher resolution to simulate and reproduce heterogeneous local scale processes.

**Figure 5:**
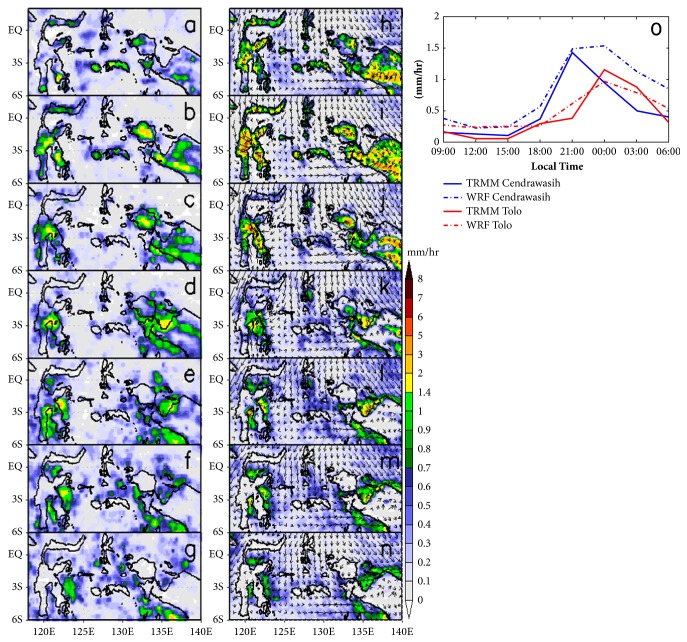
Diurnal rainfall over Maritime Continent based on TRMM 3B42 (left side) during March 2014 with (a) 12LT, (b) 15LT, (c) 18LT, (d) 21LT, (e) 00LT, (f) 03LT, and (g) 06LT. Diurnal rainfall over Maritime Continent based WRF-ARW (right side) during March 2014 with (h) 12LT, (i) 15LT, (j) 18LT, (k) 21LT, (l) 00LT, (m) 03LT, (n) 06LT, and (o) comparison of TRMM and WRF rainfall over Cenderawasih Bay and Tolo Bay.

Figures 5(a), 5(b), 5(c), 5(d), 5(e), 5(f), and 5(g) are representing diurnal rainfall cycle over IMC based on TRMM 3B42 while Figures 5(h), 5(i), 5(j), 5(k), 5(l), 5(m), and 5(n) are representing diurnal rainfall cycle over IMC based on WRF-ARW simulation. WRF-ARW can simulate the rainfall cycle with root mean square error 0.36 over Cenderawasih Bay and 0.17 over Tolo Bay. Although some events of rainfalls have overestimate, the composite diurnal rainfall cycle was simulated well and has similar phase with TRMM observation (see Figure 5(o)). The performance of all cumulus parameterization using Kain-Fritsch (KF), Betts-Miller-Janjic (BMJ), and Grell–Devenyi ensemble (GDE) schemes on WRF-ARW has been evaluated by Ratna [32]. All of the three cumulus schemes have overestimate producing rainfall bias with the KF scheme producing the largest biases.

Firstly, rainfall begins to form and grow near the mountains of the island (see Figures 5(a) and 5(b)) when the sea breeze has intense activity at daytime (see Figures 7(a) and 7(c)). Different rates of heating over inland and adjacent water cause sea breeze to flow more intensely. Since the distance and the mountain area are very close to the coastline, the sea breeze is forced to lift near mountainous area. It quickly cools down and generates condensation to develop cloud and rainfall around mountaintop. At Sulawesi Island (Tolo Bay), the rainfall concentrates around center of Sulawesi Island (mountainous area), the southeastern part of Sulawesi Island, and near Gorontalo (Mountain of Tentolomatinan, 2207 m). Over Papua Island, the rainfall is located around the middle of island (mountainous area) and western of Papua (Mountain of Bon Irau, 2501 m). This condition develops and continues until early evening (see Figure 5(c)). The effect of high orography to force local circulation inducing convective development and propagation rainfall over Papua Island was demonstrated by Zhou and Wang [33]. The orographic forcing affects diurnal rainfall through three major processes. Firstly, the terrain height increases the moisture convergence at low levels by blocking and deflecting of sea breeze. Secondly, the anabatic winds assist the initiate convection over mountaintop in the afternoon. Finally, the deep convection over mountaintop acts as a source of propagating gravity waves, which help initiate rainfalls in the coastal offshore area in the late evening to early morning.

Figures 5(h), 5(i), and 5(j) show that WRF-ARW overproduces the rainfall in both Sulawesi Island and Papua islands at daytime. The terrain effect on WRF-ARW produces a very high rainfall around mountain, although the spatial pattern of rainfall has a good pattern with the observation by TRMM 3B42. Rainfall that was produced by WRF-ARW has better agreement with TRMM 3B42 rainfall observations over flat area or lower topography [21].

At early evening (see Figure 5(d)), the rainfall over mountaintop significantly begins to move to oceans, over both Cenderawasih Bay and Tolo Bay. Cenderawasih Bay has a peak offshore intensity at early time compared to Tolo Bay (see Figures 5(d) and 5(e)). This condition indicates that the land breeze flow onset over Cenderawasih Bay is more quickly intense and stronger than land breeze flow over Tolo Bay. Surface land breeze flow generated by WRF-ARW over Cenderawasih Bay is about 0.4 m/s while over Tolo Bay it is about 0.2 m/s (see Figures 7(b) and 7(d)). The onset of land breeze flow depends on the height of the terrain and monsoonal flow. Strengthening of land breeze flow due to terrain height is tying the blocking of the sea breeze density current during the warm phase of the cycle. Blocking of sea breeze creates a pool of relatively cold, stagnant air at the base of the terrain, which in turn produces a stronger land breeze density current the following morning [24]. Ichikawa [26] also describes land breeze which produced propagation of rainfall over Papua Island depending on monsoonal flow. During boreal winter, rainfall has widespread area over land during late evening and shift to over ocean during early morning, while during boreal summer rainfall stays over the island and is persistent until midnight.

During nighttime where the land breeze has intense activity, ocean region over Cenderawasih Bay and Tolo Bay has highest offshore rainfall intensity (see Figures 5(d), 5(e), and 5(f)). Land breeze convergence is indicated as the trigger for strong convective activity over oceans (see Figures 7(b) and 7(d)). The increasing nighttime thunderstorms over Mediterranean eastern coast is associated with surface wind direction [2]. Because of the coast being concave toward sea, the land breeze constitutes a convergent wind field. This land breeze convergence makes it important to produce nocturnal thunderstorms. Figures 7(b) and 7(d) clearly show that the land breeze convergence has strong intensity over ocean during nighttime. Baker et al. [34] have simulated the effect of soil moisture and coastline curvature to rainfall; they suggest that soil moisture acts as moisture source for increasing the convectively available potential energy (CAPE) while coastline curvature shape has a significant impact on the timing and location of rainfall. Low level convergence occurs in inland near convex coastlines, and subsequent heavy rainfall occurs earlier in simulations with curved coastlines. Early-morning land breezes influence the timing of rainfall by affecting the low level convergence. WRF-ARW can simulate the offshore rainfall during nighttime very clearly. Boundary of offshore rainfall is produced by WRF-ARW quite similar to the boundary of coastline (see Figures 5(k), 5(l), and 5(m)). This condition indicates that rainfall over ocean significantly depends on shape of coastline and land breeze activity.

During early morning (see Figures 5(g) and 5(n)), dissipation of offshore rainfall begins to occur over both Cenderawasih Bays and Tolo Bay. The rainfall system becomes smaller convective cells and new offshore rainfall moves to open the ocean indicated as the squall line [30, 35]. This squall line is formed by convergence system between air cold pool wind and prevailing wind.

**Figure 6:**
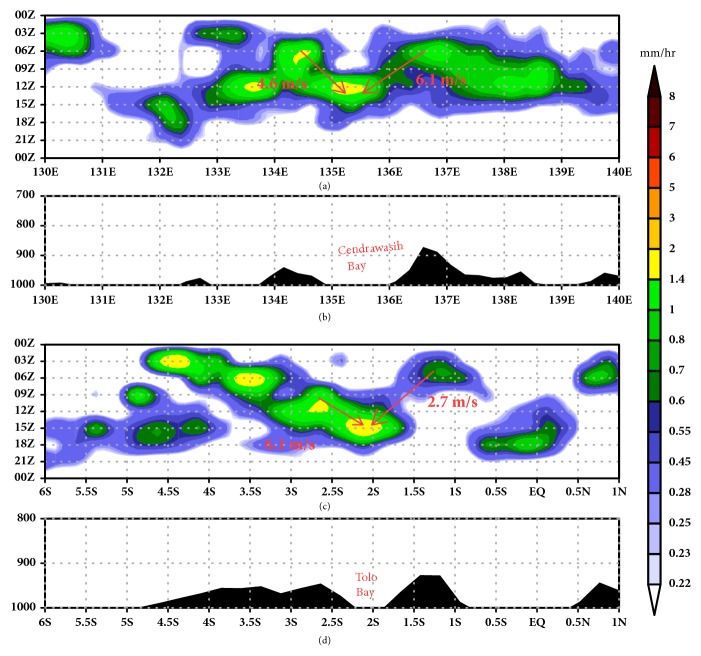
Hovmoller diagram shows propagation of rainfall over (a) Cenderawasih Bay and (c) Tolo Bay. (b) is the terrain height around Cenderawasih Bay based on cross section in Figure 2 (white line). (d) Terrain height around Tolo Bay based on cross section in Figure 2 (red line).

The migration of offshore rainfall over Cenderawasih Bay and Tolo Bay is revealed by Figures 6(a) and 6(c). The rainfall moves from the inland sides, merging in the middle of the ocean with various speeds. Rainfall over Cenderawasih Bay propagates with an average rainfall speed of around 5.4 m/s, having westerly movement about 4.6 m/s and easterly propagation approximately 6.1 m/s. The rainfall movement over Tolo Bay to ocean has an average speed around 4.1 m/s with speed of southerly movement approximately being 6.1 m/s and northerly movement about 2.7 m/s. During the movement, the peak of rainfall has a decreasing intensity although it has increased over the mountaintop during the daytime with high intensity over the ocean during the nighttime (see Figures 6(b) and 6(d)).

The speed movement rainfall over concave coastline has a slower speed than the rainfall movement from the mountaintop to open ocean. Ichikawa and Yasunari [26] explained the fact that the rainfall at New Guinea propagates faster over water than over land, with an inferred phase speed of about 7-8 m/s. Rainfall over Sumatera has a peak at the daytime and one at the nighttime migrates to coastline with average speed approximately being 10 m/s reaching the distance from the coastline up to 400 km [6]. The rainfall propagation over the Bay of Bengal has of 15–20 m/s by extending its propagation phase up to 1000 km from the coastline [11].

**Figure 7:**
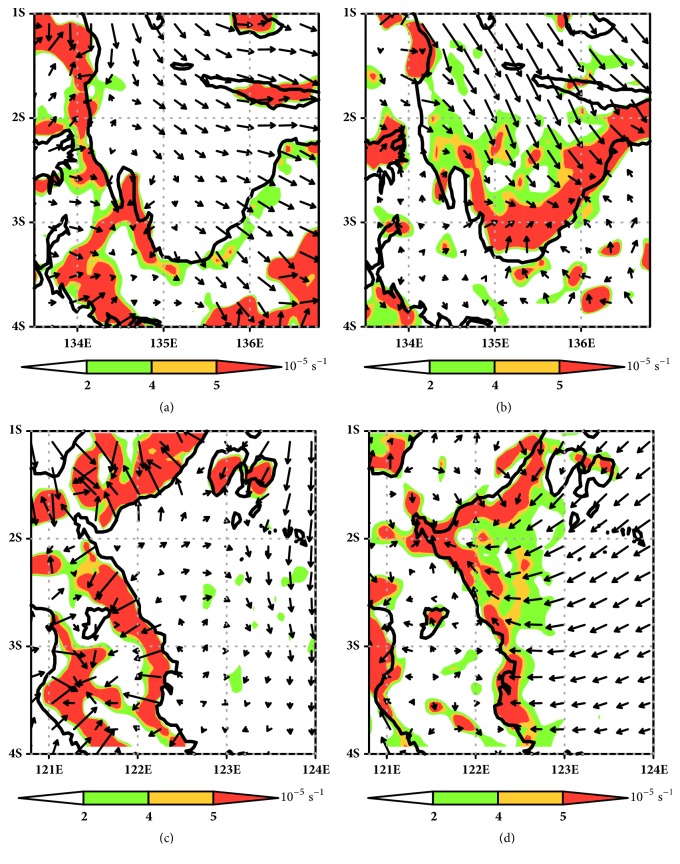
Low level convergence zone over Cenderawasih Bay during (a) daytime (12 am) and (b) nighttime and low level convergence zone over Tolo Bay during (c) daytime (12 am) and (d) nighttime.

Figure 7 reveals a shift of the convergence system from the inland (see Figures 7(a) and 7(c)) to the ocean area (see Figures 7(b) and 7(d)) both over Cenderawasih Bay and Tolo Bay. Cenderawasih Bay and Tolo Bay have the same pattern of shift convergence. Convergence occurs at inland during the daytime and shifts to the offshore area at nighttime. At the morning, the wind system does not significantly produce convergence on neither inland nor offshore. The beginning of convergence starts to form at 1200LT and is located on inland as sea breeze front [36]. Some sea breeze front located on coastal plain area can penetrate to the inner area until reaching the mountain [37]. Sea breeze front can penetrate over the inland until 400 km in the northern Australia [38], 75 km along west coast of India [39], and just few km over northern coastal plain over West Java [40].

Intense convergence over ocean occurs during the nighttime. Because of the strong land breeze flow, the convergence moves its position from the land to ocean. This convergence over ocean is formed between the land breeze and prevailing winds, facing inland direction. This mechanism also occurs in other regions such as Borneo [35] and Sumatera [16].

### 3.3. Schematic Offshore Rainfall over Concave Coastline

Previous research has suggested schematic rainfall over the IMC without considering the shape of coastline [6, 14, 16, 26]. A schematic on developing oceanic convective system in the western ocean of Sumatera that induces heavy rainfall over ocean was proposed by Trismidianto [16]. The oceanic convective system developed from midnight until the early morning, and it was intensified by the land breeze from Sumatra. New convective activities are generated with the decaying of oceanic convective system and they propagated to the western coast of Sumatra due to a divergent outflow from a cold pool. The combination of the land breeze from Sumatra and cold pool outflows from the decaying oceanic convective system was a significant factor that induced strong rainfall. Schematic offshore rainfall also suggested propagation rainfall over western coast of Sumatera [6], western coast of Borneo [6, 13, 14, 33], and New Guinea [26]. The convective activity over oceans over northwest of Borneo Island increases during monsoon surge and decreases during monsoon lulls. The offshore convective activity begins to develop around midnight when the land breeze begins and meets the monsoonal northeasterly flow [13]. All the schematic offshore rainfall suggests that the structure of rainfall propagation is squall lines.

**Figure 8:**
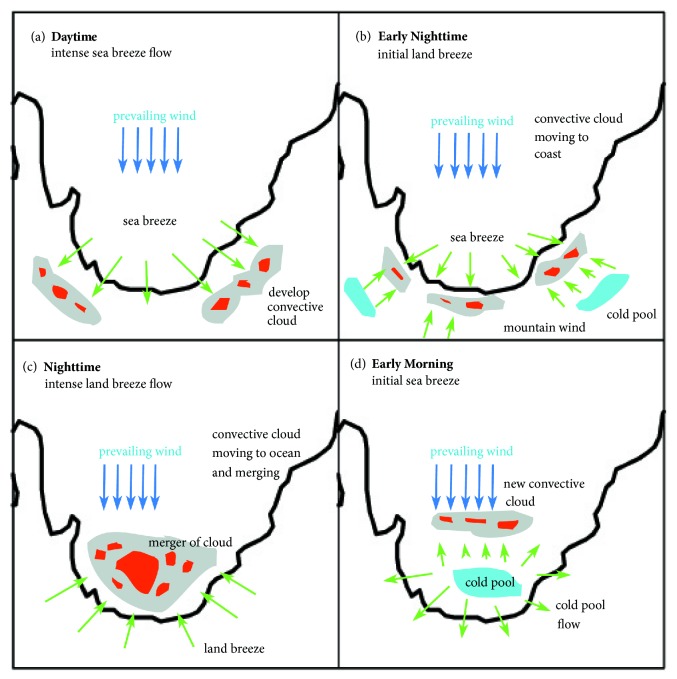
Schematic of rainfall and migration during (a) daytime, (b) early nighttime, (c) nighttime, and (d) early morning.

The dynamical atmosphere which induces the offshore rainfall over concave coastline is not quite far different from the mechanism developing offshore rainfall over west coast of Sumatera or west of Borneo Island. The main difference is the direction of land breeze due to the shape of concave coastline which creates convergence zone just only located in the middle of ocean. The merger of rainfall and propagation of rainfall from inner to outer side of ocean also causes more complex system. The mechanism of developing offshore rainfall over concave coastline can be seen in Figure 8.

Sea breeze plays a significant role in the formation of inland rainfall at daytime while land breeze plays a major role in developing offshore rainfall at night until morning time. The development of rainfall begins at inland when the sea breeze has intense wind speed (see Figure 8(a)). Because solar radiation heats the islands more effectively than the seas, sea breezes are initiated. Rainfall formed over inland area induced by topography effect. Mountain around coastline lifts the oceanic air mass to develop cloud over mountainous area.

At the early nighttime (see Figure 8(b)), mountain wind due to cold pool flow from dissipation rainfall begins to be accompanied intensely by the weakening of the sea breeze. Rainfall system propagates to costal area due to the migration of convergence zone (Figure 7(c)). The rainfall appears as squall line moving to the ocean. Squall line system propagates to the north area of the Small Island (Biak) which can induce high rainfall in Biak. The precipitation frequency from midnight to morning is large and the predominant cloud type is stratiform [30].

The highest offshore rainfall occurs during intense land breeze existence (see Figure 8(c)). Since the coastline is concave, land breeze from one and another coast side meets in the middle of ocean as low level convergence. Consequently, the rainfall propagates to the middle of ocean and induces high rainfall. The rainfall merges and develops new convective system due to this convergence generating high offshore rainfall intensity. Land breeze convergence induces offshore rainfall occurring over strait [7] and western coast of Sumatera [16]. The morning rainfall peak at the Malacca strait is induced by the convergence of two cold outflows which have been produced by the precipitation system in the previous evening over Sumatra and Malay peninsula.

**Figure 9:**
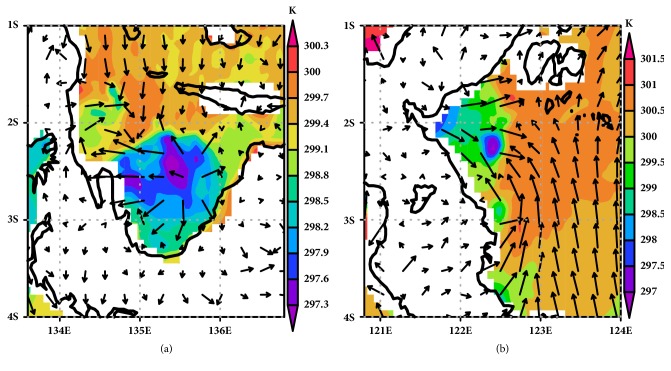
Surface potential temperature over (a) Cenderawasih Bay and (b) Tolo Bay. Lower temperature (K) is indicated as cold pool area.

In the early morning (see Figure 8(d)), the dissipation of rainfall is generated by the development of the cold pool (Figure 9). Convergence from cold pool flow and prevailing wind generate new convective system in outer side of concave coastline. The rainfall propagates from inner concave coastline to outer ocean with squall line system.

## 4. Conclusion

In this study, we found that the concave coastline is a significant factor increasing the nighttime convective activity over ocean. Concave coastline generates more intense land breeze convergence than adjacent ocean with a straight coastline. The highest mountaintop around Cenderawasih Bay and concave coastline shape compose the highest offshore rainfall over Indonesian Maritime Continent. Monthly peak offshore rainfall over concave coastline is related to direction of concave coastline and peak of diurnal cycle influenced by the shifting low level convergence. Concave coastline facing the north has peak during northwesterly monsoonal flow (March), while concave coastline facing the east has peak during easterly monsoonal flow (July). Low level convergence zone shifts from inland during daytime to ocean during nighttime. Strong land breeze induced more low level convergence intensity to produce more offshore rainfall.

The schematic offshore rainfall over IMC is a guidance weather forecaster operational in making weather forecasts especially maritime weather information. By considering the circulation of land breeze and convergence position, the weather forecaster can determine potential high offshore rainfall area.

## Figures and Tables

**Table 1:** WRF-ARW configuration for this experiment.

	Domain 1	Domain 2	Domain 3
Resolution	27 km	9 km	9 km
Parameterization			
Microphysics	WSM 6 class	WSM 6 class	WSM 6 class
Cumulus	Kain–Fritsch	Kain–Fritsch	Kain–Fritsch
Planetary Boundary Layer	YSU	YSU	YSU
Radiation	Dudhia	Dudhia	Dudhia
Land Surface	Noah LSM 4-layer	Noah LSM 4-layer	Noah LSM 4-layer

## Data Availability

The data supporting this article is provided within the article. The datasets generated and analyzed during the current study are available from the corresponding author upon reasonable request.
